# Cholecystitis: A Rare Presentation for Diffuse Eosinophilic Granulomatosis With Polyangiitis

**DOI:** 10.14309/crj.0000000000001023

**Published:** 2023-04-14

**Authors:** Nikhil Seth, Phi P. Tran

**Affiliations:** 1 Baylor Scott and White Internal Medicine, Temple, TX

**Keywords:** cholecystitis, EGPA, eosinophilic granulomatosis with polyangiitis

## Abstract

Eosinophilic granulomatosis with polyangiitis is a rare disorder of small- to medium-sized vessel vasculitis that can affect a multitude of organ systems. It typically presents as asthma, with 50% of cases having some gastrointestinal involvement, but involvement of the gallbladder is very rare. We present a unique case report of a patient who presented for nonspecific symptoms, ultimately leading to a cholecystectomy, which officially histologically diagnosed them with eosinophilic granulomatosis with polyangiitis.

## INTRODUCTION

Eosinophilic granulomatosis with polyangiitis (EGPA), formerly named Churg-Strauss syndrome, is a rare disease of small- to medium-sized vessel vasculitis, with an incidence of 1–3 cases per 100,000.^1^ EPGA is characterized by airway inflammation, blood and tissue eosinophilia, and small- to medium-sized vasculitis. The manifestation of illness is driven by eosinophil-rich inflammation and antineutrophil cytoplasmic antibodies. This disease may present as lung infiltrates, gastrointestinal (GI) involvement, and cardiac failure secondary to eosinophilic cardiomyopathy. Histologic features include necrotizing vasculitis in arteries and veins with eosinophilic infiltration in and around the vessels.^1^ GI involvement has been seen in roughly 50% of patients, with the small bowel typically being the most affected GI organ, followed by stomach and colon.^2^ Documented cases involving the gallbladder are very rare. This case highlights a unique presentation of cholecystitis that led to a clinical and pathological diagnosis of EPGA.

## CASE REPORT

**Figure 1.**
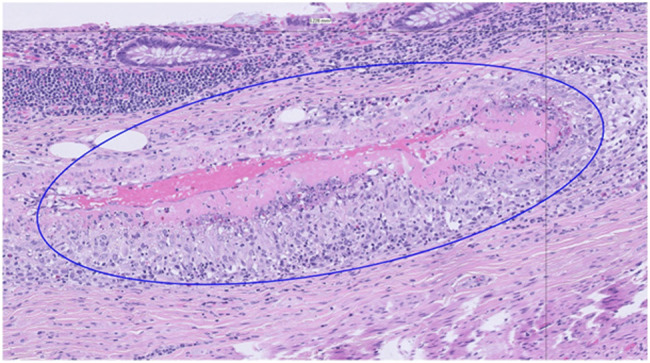
Gallbladder with eosinophilic vasculitis.

A 17-year-old girl with a history of asthma presented for 2 weeks of nausea, vomiting, abdominal pain, decreased urinary output, and 20-pound weight loss. She had a medical history significant for asthma. Physical examination was notable for an ill-appearing female patient with generalized abdominal tenderness to palpation. Laboratory test results were remarkable for a white blood cell count of 33.3 × 10^9^/L, granulocyte count of 10.46 × 10^9^/L, eosinophils of 17.44 × 10^9^/L, erythrocyte sedimentation rate 78 mmol/h, C-reactive protein 65.6 mg/L, and a urinalysis showing 20–50 red blood cells. A renal ultrasound was performed to evaluate for pyelonephritis, but incidentally found slight thickening of the gallbladder wall and biliary sludge. Surgery was consulted, and a computed tomography (CT) of the abdomen was recommended. This showed mild thickening with surrounding edema of the gallbladder. The patient persistently complained of abdominal pain and nausea. Based on her persistent pain, leukocytosis, and imaging findings, she was started on ceftriaxone, and a cholecystectomy was performed. She was steadily able to tolerate fluids and foods without nausea or pain. Despite persistently elevated white blood cell count and eosinophilia, she was discharged as symptoms improved. Gallbladder pathology returned showing necrotizing vasculitis with fibrinoid necrosis and marked eosinophilia concerning for EGPA (Figure 1).

New workup noted positivity for antineutrophil cytoplasmic antibodies. She met clinical criteria for EGPA based on history of asthma, allergies, eosinophilia, and extravascular eosinophils. With the gallbladder findings proving the official EGPA diagnosis, she was called in to follow-up. She was found to have acutely developed profound weakness in right upper and bilateral lower extremities, so she was readmitted for EGPA related peripheral vasculitis neuropathy. She was started on 1 g of solumedrol with a rheumatology consult for medication planning. Her eosinophil count improved, but she then developed worsening abdominal pain and septic shock. Abdominal x-ray showed free air in the abdomen, and CT of the abdomen showed ascites, pneumoperitoneum with concerns for perforation, and hyperenhancement of the small bowel concerning for enteritis. She was taken to the operating room and was found to have small bowel perforation and necrosis requiring resection. Her steroid dosage was decreased for concerns of infection. She then developed proteinuria with concerns of renal involvement. She was started on cyclophosphamide in conjunction to steroids. She steadily improved and was discharged on a cyclophosphamide and steroid combination for maintenance with plans for out-patient follow-up.

## DISCUSSION

**Figure 2.**
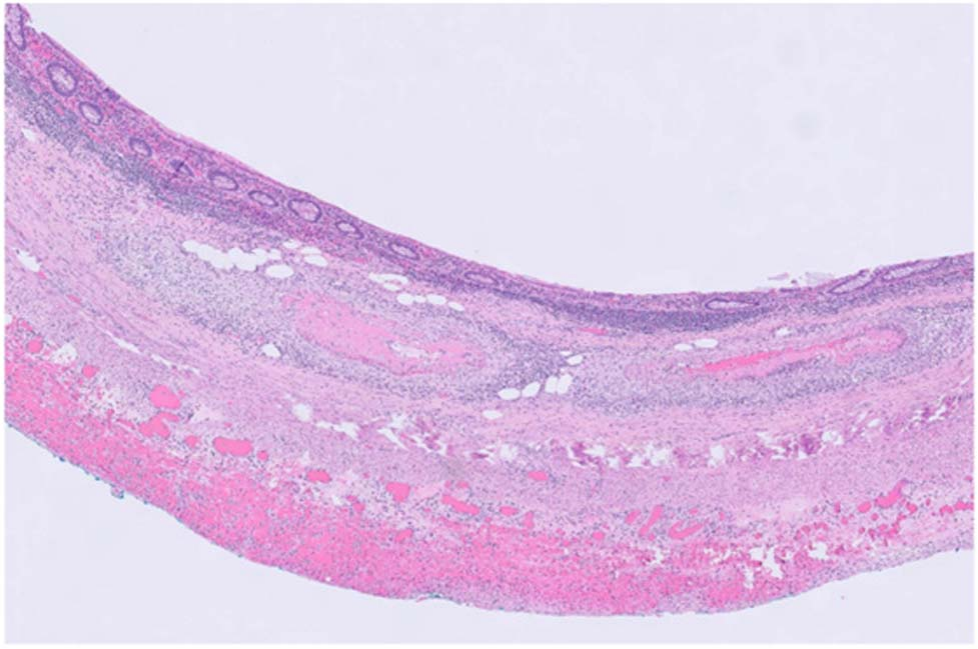
Appendiceal vasculitis.

Eosinophilic granulomatosis with polyangiitis is a rare diagnosis, and EPGA involving the gallbladder is even more uncommon. Diagnostic criteria are mostly clinical, requiring 4 of the 6 of the following: asthma, eosinophilia, history of allergy, pulmonary infiltrates that are nonfixed, paranasal sinus abnormalities, and extravascular eosinophils.^3^ As blood vessel inflammation and eosinophilic proliferation are the foundation of illness, any organ system can be involved. This patient had a history of asthma, allergies, extravascular eosinophils, and eosinophilia. She was also found to have antineutrophil cytoplasmic antibody positivity and histopathologic results of necrotizing vasculitis with fibrinoid necrosis and marked eosinophilia in the gallbladder, appendix, small bowel, and kidney. Histologically and clinically, this patient met criteria for EPGA (Figure 2).

Of all organ systems possibly affected by EGPA, the lungs represent the most commonly affected organ.^1^ GI involvement provides the worst mortality and includes risk of bowel perforation and mesenteric infarction.^4,5^ GI involvement typically presents with nausea, vomiting, and intestinal perforation.^4,6^ After our patient's biopsies from gladder pathology showed eosinophilic vasculitis, her hospital course was complicated by bowel perforation requiring exploratory laparotomy, a known complication of GI EGPA. The patient was treated with high-dose steroids, cyclophosphamide, and antibiotics, which steadily improved her clinical symptoms. Although ultimately stabilized and discharged, GI or cardiac involvement is the strongest indicator of poor prognosis.^4,5,7^ The only other poor prognostic indicator is age greater than 65 years, which this patient did not have.^8^

Biliary involvement may present as a cholecystitis-like picture, including right upper quadrant pain, nausea, vomiting, and poor oral intake. In this case, she had incidental ultrasound findings concerning for cholecystitis and CT scan confirmation. Her clinical picture of severe abdominal pain, septic picture, and persistent leukocytosis with the gallbladder wall thickening seen on imaging was concerning enough to warrant the cholecystectomy. As prognosis is poor in EPGA with GI involvement, early recognition is crucial to both mortality and morbidity. Mainstays of management include high-dose oral glucocorticoids, but remission therapy includes methotrexate, azathioprine, rituximab, cyclophosphamide, and mepolizumab.^9^ Early treatment is associated with good prognosis in early stages.^10^ Data are sparse on efficacy of requiring cholecystectomy in patients with gallbladder involvement of EPGA. As involvement is rare, further reports are needed to comment on the need of cholecystectomy. The question arises if steroid treatment may medically resolve the patient's pain and sway any unnecessary surgery. In patients with a history of asthma who present with symptoms of classic cholecystitis and have eosinophilia on laboratory findings, EPGA should be included on differential diagnosis. Radiologic, pathologic, and clinical findings are crucial to confirming the diagnosis, and prompt multidisciplinary treatment with steroids should be initiated to improve general prognosis.

## DISCLOSURES

Author contributions: Both authors are responsible for case report concept, drafting, layout, the workup of the patient and the presentation, interpretation, patient procedures, patient treatment, and article revisions and approval. N. Seth is the article guarantor.

Financial disclosure: None to report.

Previous presentation: This case report was presented at the ACG 2022 Annual Meeting; October 21-26, 2022; Charlotte, NC.

Informed consent was obtained for this case report.
